# Effects of Dual-Task Management and Resistance Training on Gait Performance in Older Individuals: A Randomized Controlled Trial

**DOI:** 10.3389/fnagi.2017.00415

**Published:** 2017-12-13

**Authors:** Bettina Wollesen, Klaus Mattes, Sören Schulz, Laura L. Bischoff, L. Seydell, Jeffrey W. Bell, Serge P. von Duvillard

**Affiliations:** ^1^Department of Human Movement Science, University of Hamburg, Hamburg, Germany; ^2^Science Department, Southwest Minnesota State University, Marshall, MN, United States; ^3^Department of Sport Science and Kinesiology, University of Salzburg, Salzburg, Austria

**Keywords:** dual-task balance training, strength training, walking, fall prevention

## Abstract

**Background:** Dual-task (DT) training is a well-accepted modality for fall prevention in older adults. DT training should include task-managing strategies such as task switching or task prioritization to improve gait performance under DT conditions.

**Methods:** We conducted a randomized controlled trial to evaluate a balance and task managing training (BDT group) in gait performance compared to a single task (ST) strength and resistance training and a control group, which received no training. A total of 78 older individuals (72.0 ± 4.9 years) participated in this study. The DT group performed task managing training incorporating balance and coordination tasks while the ST group performed resistance training only. Training consisted of 12 weekly sessions, 60 min each, for 12 weeks. We assessed the effects of ST and BDT training on walking performance under ST and DT conditions in independent living elderly adults. ST and DT walking (visual verbal Stroop task) were measured utilizing a treadmill at self-selected walking speed (mean for all groups: 4.4 ± 1 km h^-1^). Specific gait variables, cognitive performance, and fear of falling were compared between all groups.

>**Results:** Training improved gait performance for step length (*p* < 0.001) and gait-line (ST: *p* < 0.01; DT *p* < 0.05) in both training groups. The BDT training group showed greater improvements in step length (*p* < 0.001) and gait-line (*p* < 0.01) during DT walking but did not have changes in cognitive performance. Both interventions reduced fear of falling (*p* < 0.05).

**Conclusion:** Implementation of task management strategies into balance and strength training in our population revealed a promising modality to prevent falls in older individuals.

**Trial registration:** German register of clinical trials DRKS00012382.

## Introduction

For elderly people, the prevention of falling and the ability to walk safely are among the most important factors for social interaction and participation in activities of daily living (Amadori et al., 2014). Consequently, it is of great interest to foster, ensure, and when possible improve walking performance in older adults with appropriate training programs (Skelton and Todd, 2005; Sherrington et al., 2008).

Studies have reported that a stable gait pattern is primarily influenced by postural control and the control of the center of mass over the base of support (Shumway-Cook and Woollacott, 2007; Perry, 2010). The two major functions required for postural control are: (1) balance coordination (synergies in the motor cognitive system that are responsible for postural control) and (2) balance recovery, defined as the skill to gain or regain postural stability (Fraizer and Mitra, 2008; Wollesen et al., 2015, 2016).

In addition to postural control, other biomechanical or kinematical attributes have exhibited the importance of maintaining acceptable and erect posture, and furthermore, these attributes affect gait. For instance, active rolling movements of the foot and ankle joints as well as the fixation of the pelvis from heel strike to mid-stance are crucial to manage balance while moving forward (Perry, 2010). Further, the peak reaction forces, the length of the foot rolling movements (gait-line), step length, and step width were found to be paramount components that describe a stable gait pattern (Wollesen et al., 2016). Additionally, the surrounding environment influences gait. In daily life, gait is not executed in isolation, and it is more likely to be part of a dual or multitask activity whereby the walking is merged with additional cognitive or motor tasks (Faulkner et al., 2007). These dual-task (DT) conditions cause so-called dual-task costs (DTC) that are responsible for scaled down performances in one or both tasks when compared to a ST condition as a result of amplified cognitive demands (Woollacott and Shumway-Cook, 2002; Al-Yahya et al., 2010; Beurskens and Bock, 2012).

While some of the changes in walking kinematics in DT perhaps have a beneficial impact on maintaining a stable gait pattern (e.g., reduced gait speed), others (e.g., reduced step length and gait-line) may hamper and elicit less stable gait patterns (Wollesen et al., 2015). Several studies reported greater gait variability (e.g., variability of step length and speed), a lower step length, and reduced rolling foot movements with additional shift of plantar pressure in DT in older adults (Springer et al., 2006; Montero-Odasso et al., 2012; Wollesen et al., 2015). These observations have been interpreted as indicators of increased risk of falling (Woollacott and Shumway-Cook, 2002; Springer et al., 2006; Al-Yahya et al., 2010).

Dual-task costs have been found to be indicative of a decrement in cognitive performance (e.g., prolonged reaction time) that is associated with a greater risk of falling (Montero-Odasso et al., 2012). As reported by Schäfer and Schumacher (2011), elderly people tend to prioritize motor ability over cognitive tasks when balance is threatened. This has mainly been detected in standing and walking tasks (Schäfer and Schumacher, 2011). When fallers are compared to non-fallers, they show a scaled-down balance performance in DT that is indicative of reduced ability in executive functions and the focus to prioritize gait (Springer et al., 2006).

Another aspect that may result in problems to shift attention to postural control is concerns about or fear of falling, which has been identified as a common indicator of increased risk of falling. The review of Young and Williams (2015) found that a fear of falling interferes with attentional resources and the ability to appropriately acquire sensory information and therefore may impair gait in general (Young and Williams, 2015). Considering these findings, mobility training and fall prevention studies often intend to train gait performance under DT conditions (Wollesen and Voelcker-Rehage, 2013; Plummer et al., 2015).

Motor (balance or strength) or cognitive training are known to improve DT performance in older adults (Voelcker-Rehage and Alberts, 2007; Doumas et al., 2008; Hall et al., 2011). Training effects, however, are specific in a way that motor training alone improves motor function and cognitive training improves cognitive performance under DT conditions (Bherer et al., 2008; Silsupadol et al., 2009; Wollesen et al., 2015). The existing DT training studies combining motor and cognitive tasks in DT settings are promising; however, they differ in their outcomes due to their specificity and design of their training program (Wollesen and Voelcker-Rehage, 2013).

In general, DT training studies have been designed to enhance motor-cognitive DT performance while walking e.g., for persons with Parkinson disease (Fritz et al., 2015). A review by Wollesen and Voelcker-Rehage (2013) reported that healthy independently living older persons showed that DT walking performance and DTC improved after DT motor–cognitive practice with regard to cadence, gait variability, and walking speed (Toulotte et al., 2006; Silsupadol et al., 2009; You et al., 2009; Trombetti et al., 2011). Therefore, one may conclude that DT training benefits walking performance under DT conditions that reduces fall risks. These positive aspects of DT training were not yet transferred into practice of falls prevention. For example, an updated meta-analysis by Sherrington et al. (2011) does not report fall prevention as result of DT training. The authors recommended that resistance training and walking provides the most benefit to reducing fall risk when training is integrated with a balance intervention. Additionally, walking in these integrated programs should not consist of fast walking (Sherrington et al., 2011). These recommendations are consistent with the well-known benefit that single task (ST) strength and resistance training improves the gait performance of older adults (Persch et al., 2009; Hortobágyi et al., 2015).

However, in the reviews by Wollesen and Voelcker-Rehage (2013) and Plummer et al. (2015), none of the included studies examined the recommendations by Sherrington et al. (2011) or ST strength and resistance training to improve DT walking performance. Hence, it remains unclear which effects of ST resistance training can be expected to be most beneficial in DT walking performance. If falls occur in DT settings, or when walking fast, older adults need motor–cognitive strategies to reduce the risk of falling for this circumstance and ST strength training may then be less beneficial.

Moreover, it seems reasonable to regard the methods of a mobility or fall prevention training under DT conditions. Due to the positive results of some DT interventions one should include variable task prioritization and task switching elements to warrant transfer effects (Bherer et al., 2008; Silsupadol et al., 2009; Li et al., 2010; Wollesen et al., 2015). In addition, training protocols should include increasing demands with a certain minimal duration and level of task specificity to gain task-related adaptations and to optimize cognitive and motor performance (Wollesen and Voelcker-Rehage, 2013). To minimize the risk of falling and to reduce anxiety associated with falling, participants should learn task-managing strategies that allow them to switch between tasks and to prioritize the motor task to prevent instability. Integration and examination of all of these elements (task prioritization, task switching, task managing strategies) in DT training has not yet been examined.

Therefore, we developed and conducted a DT motor cognitive balance training (balance DT managing training, BDT) that included tasks designed to develop task-managing strategies related to activities of daily living to reduce DTC while walking. It is unclear whether strength and resistance training (ST) or BDT contributes further in improving DT walking performance; thus, the aim of this study was to compare these two interventions in a randomized controlled study design. Our hypothesis was that the BDT practice will improve motor DT performance while walking in association with the relevant gait variables (primary outcome) of step width, gait-line, maximum forces of the heel, mid-foot, and forefoot as well as gait variability in step width and step length. In addition, we hypothesized that resistance training will only benefit the ST walking condition. Another goal of the research was to detect the effects of the two interventions on concerns of falling (COF).

## Materials and Methods

In this randomized controlled trial study, we compared two group-based interventions (BDT to ST strength and resistance training) with the addition of control group (CG). The study was part of an ongoing research project that was approved by the Ethics Committee of the Hamburg Chamber of Physicians (PV4376). Information about the aims and risks of the study were provided to all participants in accordance with the Declaration of Helsinki. Prior to any testing, all subjects signed informed consent.

### Participants

Initially, 90 participants (mean age = 72.0 ± 5.0 years of age) were recruited to account for statistical mortality rate. The results of an a priori sample size calculation [G^∗^power 3.1., analysis of variance (ANOVA): repeated measures within-between interaction factors; *f* = 0.25; with alpha error probability of 0.05; power 0.95, number of groups 3; number of measurements = 2]. Sixty-six participants were necessary for appropriate statistical power.

The recruitment was conducted via the use of advertisements in local newspapers. The inclusion criteria were: living independently; 70–80 years of age, and the ability and mobility to join the group training sessions. According to previous study design used in this research project (Wollesen et al., 2015) exclusion criteria were: acute or chronic disease with documented influence on balance control (e.g., Parkinson’s disease; diabetes or peripheral neuropathy), use of assistive gait devices (e.g., walking canes and frames), and a Mini-Mental Status Examination (MMSE) of less than 25 to exclude any cognitive impairment. Participants who participated in moderate exercise were excluded to avoid confounding factors that could skew or influence the main outcomes of our study. A senior researcher, who was not part of the initial research group randomly grouped the 90 participants using a program provided by www.randomizer.org. Randomization was stratified for the scores on the Short Physical Performance Battery (SPPB), sex, and age. **Table 1** depicts the physical characteristics of the participants (*N* = 78).

**Table 1:** Physical characteristics of the 78 participants.

Variables	Balance and task-managing training (*n* = 29)	Strength and resistance training (*n* = 23)	Control group (*n* = 26)
Age (years, *SD*)	70.7 (4.9)	71.7 (4.9)	73.7 (5.0)
Females (%)	22 (75.9)	15 (65.2)	19 (73.1)
Males (%)	7 (24.1)	8 (34.8)	7 (26.9)
Height (cm; *SD*) females	168.6 (6.3)	163.8 (6.2)	164.4 (6.5)
Height (cm; *SD*) males	179.8 (7.2)	177.9 (5.4)	177.4 (7.2)
Weight (kg; *SD*) females	72.1 (10.1)	69.0 (12.6)	74.1 (10.9)
Weight (kg; *SD*) males	85.7 (10.5)	83.0 (4.5)	84.1 (4.3)
SPPB (score; *SD*)	10.9 (1.0)	11.1 (1.0)	11.1 (1.0)
Walking speed (km h^-1^; *SD*)	4.3 (1.0)	4.27 (1.0)	4.6 (1.0)
MMSE	27.2 (2.3)	26.8 (2.9)	27.4 (2.6)
FES-I (score; *SD*)	20.0 (3.0)	21.1 (5.2)	18.6 (2.2)

*Values are mean (±SD) or percent (%). FES-I, falls efficacy scale international; MMSE, mini-mental status exam; SPPB, short physical performance battery; SD, standard deviation.*

**FIGURE 1:**
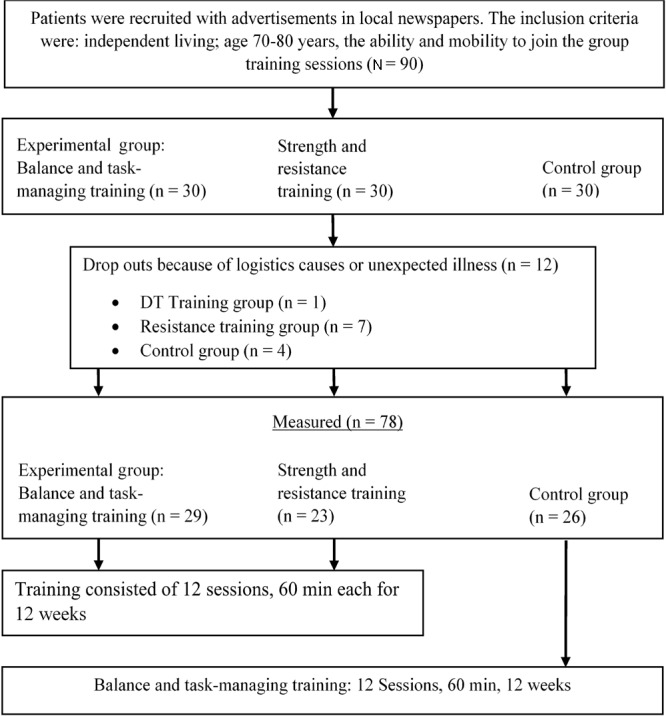
Study flow.

Analysis of physical characteristics and health-related data, as well as fear of falling (FES-I), yielded no statistical significance (*p* > 0.05) between groups. There were 12 drop-outs (one in the BDT training group; seven in the strength and resistance training group, and four in the CG). The main causes for drop out were logistics (travel time, travel means, transportation, etc.) and unexpected illnesses. **Figure 1** shows the study flow.

### Description of the Intervention

#### BDT Training

The standardized BDT training program followed the intervention described by Wollesen et al. (2015). It consisted of two phases with increasing demands. Instructors received specific information, training, and needed to be qualified and certified for the program.

#### Phase (1) Training of Daily Actions with a Likelihood of Fall Risk (Weeks 1–6)

In the first phase, activities and conditions mimicked those that would be encountered in daily life and could potentially be linked to a higher fall risk during walking (e.g., brisk walking, starting, stopping, avoiding obstacles, sidesteps, turns).

Explanations were provided to introduce awareness of trip hazards, speeds required, and additional tasks (including visual and proprioceptive) by repeating the same task several times in different task settings (e.g., carrying out the tasks with reduced base of support). They also had to negotiate balance disturbances or additional DT (motor or cognitive with visual stimuli) as described by Wollesen et al. (2015). Additionally, they were encouraged to focus on active rolling movements of their feet to maintain a stable gait pattern. The instructor educated participants about task managing strategies to assist them in recovering their balance during disturbances. These strategies are detailed below:

• “if there are obstacles in your way, try to recognize the whole area around you and your feet”
• “if there are obstacles in your way, it is better to use a foot rolling movement, because that way you will not trip over the obstacle”
• “if you recognize an increased postural sway, increase your base of support”
• “if you recognize that you may lose your balance, increase your base of support”
• “if you recognize that you may lose your balance, use a side step to recover”
• “if you recognize that you may lose your balance, bend your knees to recover”
• “if you recognize that you may lose your balance due to added tasks, focus your attention on your balance or walking performance”
• “if you are engaged in a difficult secondary task try to find a task prioritization solution (e.g., if you need to look up at a signs to find your way, try to stop and slow down, view the sign and continue walking afterward)”
• “if you are engaged in a difficult secondary task, try to switch between the cognitive and the motor component to stay aware of your walking or balance performance”
• “if you need to do a brisk walking task, try to remember your foot rolling movements”
• “if you need to stop your walking quickly, try to include balance recovery strategies like bending your knees”
• “if you are engaged in a situation with many people around you (e.g., at a train station) try to focus on your own movements and balance control strategies when someone bump into your shoulder”
• “if you have concerns about falling try to stay relaxed and bend your knees a bit to reduce muscle stiffness”

#### Phase 2: Training of Task Prioritization, Task-Switching, and Transfer (Weeks 7–12)

The second phase included tasks of greater complexity: all tasks from the first phase had to be completed under DT conditions combined with precision tasks, time pressure, task prioritization, and task switching. A simulation of daily-life situations increasingly required subjects to use task managing strategies, as described above.

### Strength and Resistance Training (ST)

The strength and resistance training followed the program of Becker et al. (2006). It included the progressive training of relevant muscle groups used in daily activities (e.g., walking, standing, or rising from a chair with wrist weights, dumb-bells, and elastic bands). The participants started with either weights or elastic bands that provided manageable resistance for them to be able to repeat the activity 10–12 times. Every fourth week, the individual subject’s progression was re-assessed and the weights and elastic bands were adapted to suit their new resistance for 10–12 repetitions.

Over a time-span of 12 weeks, the practice groups took part in 12 1-h sessions of group exercises. This design was selected because of the financial reimbursement practice of the German Health Insurances. Prevention courses that are offered and reimbursed by German Health Insurance companies cannot exceed 12 sessions, one session per week with a 60–90 min duration for each session. Due to funding, other particular criteria had to be met, ensuring the quality of this prevention program (i.e., quality of the exercise training, standardization of the program, and qualification of the practicing supervisors) (GKV-Spitzenverband, 2010). No other co-intervention or cognitive training was undertaken in addition to the DT and ST training. There was no matching of motor components in the groups except for rising from a chair that was part of training in the BDT group with a secondary task beginning at week 9. Subject compliance was monitored and recorded via attendance lists. All participants listed in **Table 1** took part in at least 10 of the 12 sessions.

### Control Group

The CG did not receive any intervention during the study; however, the CG was assured that they would be included in a new intervention after the training groups completed the ongoing study.

### Test Instruments and Measurements

#### Walking Test under ST and DT Conditions

All measurements described below were conducted at baseline (pre-test, t1) and after 12 weeks (post-test, t2) for all groups. Subjects performed a 30 s walking test at self-selected constant speed on an h/p/cosmos motorized treadmill (Zebris; Isny, Germany: FDM-T). This treadmill has built in sensors to measure peak plantar pressure and other gait kinematics. Self-selected walking speed was determined via a staircase method with walking up to a certain level of comfortable speed with increasing and decreasing speed until a comfortable pace was achieved. Before the test sessions started, all subjects practiced treadmill walking. With familiarization periods of about 5 min, participants were allowed to practice until they felt comfortable with the training device.

After the practice period, the participants were asked to signal when to commence the measurements. They did not stop walking to avoid transition from standing to walking. The individual self-selected walking speed from the baseline measurement was used to observe differences of gait variables from pre- to post-testing. The gait variables included step length, step width, and gait-line [length of the foot rolling movements (centimeter)] as well as the vertical maximal impact [peak plantar pressure of heel, mid-foot, and forefoot; determined for absolute peak pressure (N cm^2^) and normalized to body weight (N⋅cm^2^⋅kg^-1^)] were measured as main outcome variables. All gait data were collected for both feet at 100 Hz. Average of the steps over 30 s for the observed walking variables was determined by FDM-T software.

In the DT condition, subjects performed a visual–verbal Stroop test with 16 incongruent color words (e.g., the word “blue” presented in yellow letters) each within one 30 s trial per condition (familiarization, ST, walking). Stimuli were projected onto a white wall 2 m away from the participants for all conditions. Instead of naming the actual word spelled by the letters presented, they had to name the color of the font. The time interval between word insertions ranged between 0.8 and 1.2 ms to avoid rhythm of occurrence. The tests varied the sequence of word colors. Each test lasted 30 s and the overall length matched the length of the ST or DT sequence. Under ST conditions, participants performed the test while sitting. During the Stroop test while performing walking, the words were displayed at a size of 40 to 58 cm × 20 cm. To prevent a learning effect, three varying versions of the Stroop test were conducted where congruent and incongruent stimuli were presented in a randomized order. The different versions of the Stroop test (familiarization, ST, walking) were presented in no specified order. All Stroop tests were recorded on video. The video included the participant’s verbal response to the color word on the screen. The number of accurate answers was monitored, recorded, and analyzed (e.g., the word “red” was presented in blue color and the participant answered blue or the word was “red” and was presented in red color and the participant answered red). The analysis was based on all stimuli, irrespective of the congruency of the stimuli. The methods have been confirmed in previous experiments to ensure they were valid for this target group (Wollesen et al., 2016). No participant reported problems observing the words during the Stroop test.

##### Test Instructions

All participants were familiarized with a standardized protocol. The following is a translation of the instructions that were read aloud by the investigator:

“In our laboratory we will conduct two different tests. One of them is an attention test that you will complete while sitting, the other is a walking test that will be completed one time with and one time without the attention test on a treadmill. To start please take a seat on the chair.”

(1) Seated Stroop-Test

“To start, you will get one attempt to practice a color-word test called a Stroop test. The task is to name the color of a word that appears on the screen. Several names of colors will appear on a screen but in different colors. Your task is to name the color of the word aloud and not to read the word. You will wear a microphone around your neck. The test will last 30 s and you will begin after you have the opportunity to practice.”

(2) Treadmill Walking

“We will collect data during your walk on the treadmill. First, you will have the opportunity to walk at least 5 min on the treadmill until you feel secure and comfortable and you can determine a comfortable walking speed. Please try to walk as normally as you can. The speed of the treadmill will be adjusted until you achieve a comfortable walking speed. You will wear a safety belt during the test to keep you safe. The treadmill can be stopped by you or the investigators by pushing the red safety button. After you have the opportunity to practice, you will walk at the speed you selected for 30 s while keeping your head upright and your eyes focused on the white screen in front of you. The investigator will give you a start signal at the beginning of the measurement and a stop signal at the end of 30 s.”

(3) Treadmill Walking Stroop-Test

“In the last test, you will do the same Stroop test that you already completed while sitting, only this time you will do it while walking. You will walk at the same speed as you did in the previous walking test. This time the words will appear on the screen in front of the treadmill. The test will last 30 s. The investigator will give you a start signal at the beginning of the measurement and a stop signal at the end of 30 s.”

### Other Measurements

Participants were not instructed to prioritize their gait patterns or the cognitive task. After the trial, participants of the intervention groups were asked if they prioritized the cognitive or the motor task or if they used a strategy. There were six questions that could have been answered with “yes” or “no” and one open strategy question. We further inquired as to the reason why they chose the response and the reason for electing their focus.

The 16-Item Falls Efficacy Scale-International (FES-I; German language version) was applied to account for the COF. This is based on the operational definition of the fear as “low perceived self-efficacy” to avoid falls during essential, non-hazardous activities of daily living (Yardley et al., 2005).

### Statistical Analyses

For statistical analyzes we used SPSS ver. 22 computer software (IBM statistics Armonk, NY, United States). To determine differences between the task conditions (ST vs. DT, as well as the different training groups) ANOVA was calculated after all assumptions were checked (homoscedasticity, normal distribution). For the analysis of Stroop test performance (percentage of correct answers) repeated assessments were calculated via 3-way repeated measures ANOVA with either ST Stroop performance or DT Stroop performance while walking as a factor. To evaluate the pre–post effects of the walking performance (primary outcome) under ST and DT conditions, we applied ANOVA with ST and DT performance as a repeated measures factor for each calculated variable (step width, step length; gait-line; peak forces of forefoot, mid-foot, and heel). Main effects for factor, ST/DT, and the interaction of factor ^∗^ group were determined, as well as between subject effects for all groups. Significance was set at α = 0.05; normal distribution was confirmed via the Kolmogorov–Smirnov test. Effect size is presented as partial eta square ($\eta_{p}^{2}$; small effect $\eta_{p}^{2}$ ≥ 0.08, moderate effect $\eta_{p}^{2}$ ≥ 0.20, and $\eta_{p}^{2}$ ≥ 0.32 large effect ([33, 34]). A Bonferroni *post hoc* test was applied for all comparisons. The task-prioritization strategy evaluation analyzed frequencies of “yes” or “no,” as well as qualitative evaluation of the open-ended question.

## Results

**Table 2** depicts the main effect of each dependent variable. In addition, the interaction between time and group for both ST and DT conditions is presented.

**Table 2:** Primary outcome pre–post group comparisons of gait variables (*n* = 78).

Gait variable		Single-task pre-to-post						Factor pre–post			Factor ^∗^ group ST		
		BDT		Strength and resistance training		Control		*F*_(1,73)_	*p*	$\eta_{p}^{2}$	*F*_(2,73)_	*p*	$\eta_{p}^{2}$
		Pre	Post	Pre	Post	Pre	Post						
Step width (cm)		12.73 (2.8)	11.27 (3.0)	12.59 (3.9)	11.45 (4.4)	11.37 (3.9)	10.01 (2.8)	25.25	**<0.001**	0.257	0.42	0.660	0.011
Step length (cm)	*l*	38.50 (8.6)	45.68 (7.9)	40.14 (9.9)	40.67 (11.5)	49.19 (9.7)	48.45 (8.8)	15.91	**<0.001**	0.179	18.57	**<0.001**	0.337
	*r*	38.16 (9.5)	44.81 (8.5)	39.18 (10.6)	39.39 (11.9)	48.9 (11.6)	48.16 (10.2)	10.45	**<0.01**	0.125	14.11	**<0.001**	0.279
Gait-line (mm)	*l*	178.54 (45.1)	198.82 (33.7)	190.36 (37.2)	184.72 (42.6)	223.04 (43.9)	228.96 (42.9)	5.16	**0.026**	0.066	6.51	**<0.01**	0.151
	*r*	182.64 (47.6)	204.57 (33.3)	189.72 (39.7)	186.08 (45.3)	227.44 (40.6)	231.57 (43.2)	5.04	**0.028**	0.065	5.47	**<0.01**	0.130
Peak pressure forefoot	*l*	0.38 (0.1)	0.40 (0.1)	0.39 (0.1)	0.43 (0.1)	0.35 (0.1)	0.35 (0.1)	4.63	**0.035**	0.060	1.980	0.145	0.051
(N⋅cm^2^⋅kg^-1^)	*r*	0.41 (0.1)	0.43 (0.1)	0.42 (0.1)	0.44 (0.1)	0.36 (0.1)	0.34 (0.1)	0.74	0.394	0.010	1.22	0.301	0.032
Peak pressure midfoot	*l*	0.23 (0.1)	0.23 (0.1)	0.23 (0.1)	0.22 (0.1)	0.24 (0.1)	0.24 (0.1)	0.48	0.491	0.007	0.16	0.212	0.042
(N⋅cm^2^⋅kg^-1^)	*r*	0.22 (0.1)	0.24 (0.1)	0.22 (0.1)	0.22 (0.1)	0.24 (0.1)	0. 25 (0.1)	2.24	0.139	0.030	1.05	0.357	0.028
Peak pressure heel	*l*	0.25 (0.1)	0.30 (0.1)	0.28 (0.1)	0.28 (0.1)	0.30 (0.1)	0.29 (0.1)	5.77	**0.019**	0.073	6.77	**<0.01**	0.156
(N⋅cm^2^⋅kg^-1^)	*r*	0.25 (0.1)	0.30 (0.1)	0.27 (0.1)	0.28 (0.1)	0.29 (0.1)	0.30 (0.1)	10.26	**<0.01**	0.123	4.31	**0.017**	0.106

**Gait variable**		**Dual-task pre–post**						**Factor pre-post**			**Factor ^∗^ group DT**		
		**BDT**		**Strength and resistance training**		**Control**		***F*_(1,73)_**	***p***	**$\eta_{p}^{2}$**	**F_(2,73)_**	***p***	**$\eta_{p}^{2}$**
		**pre**	**Post**	**Pre**	**Post**	**Pre**	**post**						

Step width (cm)		12.14 (3.1)	11.54 (3.4)	12.34 (3.7)	11.7 (4.1)	11.53 (3.5)	10.9 (3.6)	9.74	**<0.01**	0.118	0.00	0.996	0.000
Step length (cm)	*l*	38.52 (9.3)	45.90 (7.8)	41.0 (10.5)	41.33 (11.6)	48.82 (8.8)	48.83 (8.4)	20.22	**<0.001**	0.217	18.64	**<0.001**	0.338
	*r*	37.83 (10.0)	44.45 (9.1)	39.9 (10.7)	39.53 (11.9)	48.98 (11.6)	48.66 (10.5)	8.60	**<0.01**	0.105	12.53	**<0.001**	0.255
Gait-line (mm)	*l*	187.14 (36.2)	201.71 (33.2)	196.36 (28.6)	192.0 (36.6)	229.13 (41.8)	232.3 (40.4)	2.94	0.091	0.039	4.73	**0.012**	0.115
	*r*	191.29 (36.8)	206.39 (29.7)	192.36 (35.6)	189.92 (44.3)	234.52 (35.0)	233.26 (43.1)	1.82	0.182	0.024	4.26	**0.018**	0.105
Peak pressure forefoot	*l*	0.36 (0.1)	0.39 (0.1)	0.38 (0.1)	0.40 (0.1)	0.34 (0.1)	0.34 (0.1)	3.20	0.078	0.042	2.22	0.116	0.057
(N⋅cm^2^⋅kg^-1^)	*r*	0.38 (0.1)	0.41 (0.1)	0.40 (0.1)	0.41 (0.2)	0.34 (0.1)	0.33 (0.1)	1.11	0.295	0.015	1.75	0.182	0.046
Peak pressure midfoot	*l*	0.24 (0.1)	0.25 (0.1)	0.23 (0.0)	0.23 (0.1)	0.24 (0.1)	0.25 (0.1)	4.86	0.031	0.062	0.05	0.608	0.014
(N⋅cm^2^⋅kg^-1^)	*r*	0.22 (0.1)	0.23 (0.1)	0.22 (0.0)	0.22 (0.1)	0.24 (0.1)	0.26 (0.1)	4.22	**0.043**	0.055	0.750	0.476	0.020
Peak pressure heel	*l*	0.27 (0.1)	0.31 (0.1)	0.30 (0.1)	0.30 (0.1)	0.29 (0.1)	0.30 (0.1)	4.89	**0.030**	0.063	3.835	**0.026**	0.095
(N⋅cm^2^⋅kg^-1^)	*r*	0.26 (0.1)	0.30 (0.1)	0.29 (0.1)	0.29 (0.1)	0.29 (0.1)	0.30 (0.1)	4.24	**0.043**	0.055	1.953	0.149	0.051

### Comparison of Walking Performance under Single-Task Conditions of the Training Groups

As reported in **Table 2** under ST conditions, ANOVA revealed significant main effects for reduced step width (*p* < 0.001), increased step length (*p* < 0.01), and improved gait-line (*p* < 0.05).

For the step length and the gait-line group, differences were found for the pre–post condition. The BDT group showed the largest increase in step length for both feet (BDT training left: +7.18 cm, right: +6.65 cm; strength and resistance training left: +0.54 cm, right: +0.21 cm; control left: -0.74 cm, right: -0.74 cm; *p* < 0.001) with accompanying and improved gait-line (BDT left: +20.28 mm, right: +21.93 mm; strength and resistance training left: -5.64 mm, right: -3.64 mm; control left: +5.92 mm, right: +4.13 mm; *p* < 0.01). *Post hoc* comparisons revealed significant group differences for the BDT group compared to the CG (*p* < 0.05) as well as for the strength and resistance training group and the CG (*p* < 0.05) for the step length and the gait-line of both feet. In addition, these changes were accompanied with a significant change (*p* < 0.01) in peak planter pressure of the heel for both feet (**Table 2**; BDT left: +0.05 N⋅cm^2^⋅kg^-1^, right: +0.05 N⋅cm^2^⋅kg^-1^; strength and resistance training left: no changes in right: +0.1 N⋅cm^2^⋅kg^-1^; control left: -0.1 N⋅cm^2^⋅kg^-1^, right: +0.1 N⋅cm^2^⋅kg^-1^).

### Comparison of Walking Performance under Dual-Task Conditions of the Training Groups

The same main effects were observed in ST conditions as in DT conditions for step width (*p* < 0.05) and step length (*p* < 0.01; **Table 2**). Step width was significantly reduced in all groups. Step length increased for the two intervention groups with a larger increase for the BDT group (BDT left: +7.38 cm, right: +6.62 cm; strength and resistance training left: +0.33 cm, right: -0.37 cm; control left: +0.01 cm, right: -0.32 cm; *p* < 0.001). This was accompanied by a lengthened gait-line as observed under pre–post-ST conditions (BDT left: +14.57 mm, right: +15.1 mm; strength and resistance training left: -4.36 mm, right: -2.44 mm; control left: +3.17 mm, right: -1.26 mm; *p* < 0.01). In addition, the DT condition led to increased peak planter pressures of mid-foot in all groups (*p* < 0.05; **Table 2**). For plantar pressure of the left heel a significant difference between groups (*p* < 0.05) was observed with the largest increase in the BDT group (left + right: +0.4 N⋅cm^2^⋅kg^-1^).

### Main Differences between Single and Dual-Task Walking Conditions

There was a significant group × time × ST/DT effect for step width. The BDT group reduced the delta between ST and DT from 0.6 in the pre-test to 0.2 in the post-test whereas the strength and resistance group had a delta of 0.2 in pre- and 0.1 in post-testing. The CG increased the delta from 0.1 (pre) up to 0.8 in post-testing (*F* = 6.059; *p* = 0.016, $\eta_{p}^{2}$ = 0.077).

Significant differences for the ST/DT condition were found for the gait-line (left: *F* = 8.9; *p* < 0.01, $\eta_{p}^{2}$ = 0.109; right: *F* = 4.66; *p* < 0.05, $\eta_{p}^{2}$ = 0.060). In addition, for the gait-line right, a time × ST/DT effect was found. As reported in **Table 2** the gait-line showed greater improvements for ST conditions compared to DT conditions (*F* = 3.849; *p* < 0.05, $\eta_{p}^{2}$ = 0.05). Moreover, significant differences for the ST and DT conditions were found for the plantar pressure of the forefoot (left: *p* < 0.001, right: *p* < 0.001), the left mid-foot (*p* < 0.05), and the left heel (*p* < 0.05) with no significant group differences. For all participants, the plantar pressure increased from ST to DT (**Table 2**).

### Cognitive Performance under Single and Dual-Task Conditions and Task Managing Strategies

**FIGURE 2:**
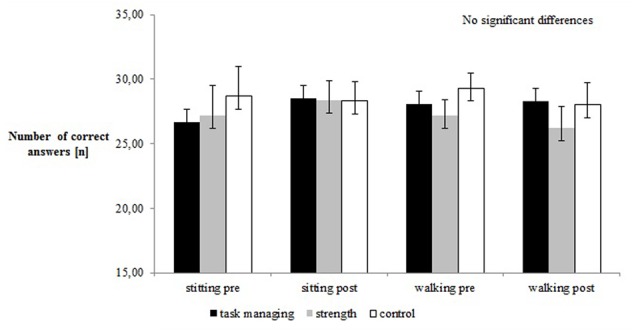
Pre–post comparison of cognitive performance (%).

**Figure 2** shows the cognitive performance for all groups. The BDT group improved their cognitive performance from pre- to post-test; however, the differences were not statistically significant. Overall, no statistically significant group differences were found for task prioritization.

As reported, the main focus of the participants in the intervention groups was on cognitive performance. Subjects reported strategies like “focus on the color,” “focus on the last letter,” and “try to concentrate.” Changes in strategy were observed in 45% of subjects in the BDT group and 55.6% of subjects in the strength-training group. The most commonly reported strategy change occurred in switching from the cognitive focus to no task prioritization. No participants reported changing the focus to gait performance.

### Concerns of Falling (COF)

A significant main effect for a reduction of COF was found (*F*_(1,66)_ = 4.98; *p* = 0.029; $\eta_{p}^{2}$ = 0.07). The BDT group and the strength and resistance group reduced COF (BDT: pre 20.0 ± 3, post 19.1 ± 2.3; strength and resistance training, pre 21.1 ± 5.2, post 20.0 ± 3.7; control: pre 18.6 ± 2.2, post 18.5 ± 2.5).

## Discussion

The aim of this randomized controlled trial was to compare the effects of two different training protocols (1) a DT balance training intervention with task managing strategies and (2) a ST strength and resistance training on motor performance during DT walking.

The overall purpose of the intervention was to reduce fall risks in older adults by improving gait performance during the cognitive demanding Stroop test. Common kinematic walking variables (e.g., step width, step length, gait-line) and kinetic gait variables (peak plantar pressure) in ST and DT conditions were determined. These variables describe an active use of the ankle joint and foot rolling movements that represent significant factors to compensate for gait disturbances and to preserve postural control (Perry, 2010).

The results of this study revealed significantly increased step length in both intervention groups after 12 practice sessions accompanied with a reduced step width in ST and DT condition, while the CG reduced their step width only. Concomitantly, the cognitive performance did not change from pre- to post-testing. This suggests that walking performance improved whereas cognitive performance remained unchanged. Therefore, one may conclude that training frees up motor resources in DT conditions. Moreover, the gait data showed the greatest improvements for the BDT training group. These results confirm our hypothesis in favor of DT training over ST training for motor improvements. Previously published investigations regarding falls and fall risks suggest increased step length may serve as an indicator for reduction in fall risk (Woollacott and Shumway-Cook, 2002; Springer et al., 2006; Al-Yahya et al., 2010). However, the reported results of this study also indicate a more stable gait pattern in ST conditions. Similar to our previous feasibility study of this training protocol (Wollesen et al., 2015) we found a reduced step width, increased step length, increased gait-line, and a more pronounced heel-strike that can be interpreted as an improvement of the foot rolling movements with a more balanced and less accident-sensitive gait pattern as reported by Perry (2010).

Additionally, our results indicate benefits of specific DT training on gait performance [e.g., for participants with Parkinson’s disease (Yogev-Seligmann et al., 2012)] and strategies of task switching and task focus as reported by Li et al. (2010).

Furthermore, in comparison to slightly younger individuals (60–70 years), elderly participants (above 70 years) tend to use increased stride length and cadence to manage the same gait speed (Wollesen et al., 2016). Modifications like these commonly co-occur with increasing maximal plantar foot pressure. To control for confounding factors in or study in DT performance, the peak plantar foot pressure was recorded at self-selected preferred speed and normalized to body mass as reported by Wollesen et al. (2016). The data presented in **Table 2** depict instances of the peak plantar pressure in mid-foot and the heel. Although, descriptively, the change from pre-to-post testing was observed in all groups but the $\eta_{p}^{2}$ was less than 0.2. Since the main effect for the group by time interactions was significant (*p* < 0.05), one may argue that the increased peak forces of the heel represents an improvement following the intervention. As shown in **Table 2**, only the BDT group increased the plantar pressure of the heel significantly. The intervention included training by intentionally training the rolling of the foot movement from heel to toe. In combination with the results of the gait-line, the gait characteristics of the balance and task managing training group exhibited greatest improvements on stabilizing their gait patterns in DT conditions. We postulate that this aspect is important for improving walking performance in older adults. With a pronounced heel strike, the whole movement of a gait cycle can be altered. This movement is task specific and needs to be trained with task-specific exercises (Yogev-Seligmann et al., 2012; Wollesen and Voelcker-Rehage, 2013). Our general strength training did not include this specificity and it may not lead to the same improvements as the task managing training.

Additionally, as a result of the reduced resources allocation model of Kahnemann (1973) in DT conditions an increased awareness is needed to comparably manage both tasks. This outcome in motor DT performance may be interpreted as a decrement in one of the tasks. Since there was no change in cognitive performance (**Figure 1**), the intervention groups may have improved their allocation of resources for motor performance in DT conditions. This hypothesis may need to be verified with neuroimaging in future studies.

The BDT intervention group demonstrated significant improvements in relevant gait patterns in DT conditions. Since walking with a visual–verbal Stroop task was not part of the training intervention, one may deduce that these improvements are resulting from the exercise program that contributed to the benefits of the BDT group. Nevertheless, the duration of the intervention has to be extended to determine whether the differences between the two interventions may also be increased. An increased training duration may amplify the benefits of the task managing training in comparison to the ST strength training while DT walking and may also improve cognitive performance. Another reason for the increased performance of the BDT intervention group may be that the intervention improved DT managing strategies. However, following the premise that fallers are not able to prioritize gait performance (Schäfer and Schumacher, 2011), the balance and task managing program was intended to introduce this task switching from a cognitive task to a walking task. Unfortunately, the intervention conducted in this study did not accomplish this objective. We observed a shift in focus from the cognitive task to lack of specific focus to either the cognitive or the motor task in the intervention groups (**Table 3**). However, the DT training group did not focus their walking performance as expected. A partial explanation for the observed results was revealed by the qualitative response by some of the participants that they were more interested in the presented cognitive task. Following the task prioritization model of Yogev-Seligmann et al. (2008) one may advocate that the laboratory environment was not sufficiently challenging to require a switch from the cognitive focus to motor control. This was confirmed by our results presented in **Table 3**. Thus, future studies may wish to include a more challenging walking task so the equalization between the BDT intervention may be achieved and therefore be transferable to activities of daily living. In addition, the COF in our group of participants cannot be rated as elevated or superior (Delbaere et al., 2010). The results of our study suggest that the participants were not forced to prioritize gait as they felt quite confident in managing the DT. The interventions did, however, reveal benefits and reduction in FES-I score for participants with COF.

**Table 3:** Evaluation of the task managing strategies of both groups.

Question	DT balance and managing training				Strength and resistance training			
	Pre		Post		Pre		Post	
	Yes%	No%	Yes%	No%	Yes%	No%	Yes%	No%
Did you feel insecure while standing with the addition of Stroop task?	10.5.	89.5	10.5	89.5	7.7	92.3	0	100
Did you feel insecure while walking with the addition of Stroop task?	15.8	84.2	10.5	89.5	0	100	12.5	87.5
I was annoyed by my mistakes during the Stroop task	50	50	40	60	53.8	46.2	50	50
Did you concentrate on the Stroop task and not on gait performance?	70	30	75	25	76.9	23.1	71.4	28.6
Did you try to apportion attention to both task equally?	46.4	54.6	60	40	84	16	62.5	37.5
Was the walking speed too fast while walking during the Stroop task?	5	95	5	95	0	0	0	0
DT strategy focus on cognitive performance	55	45	40	60	33.3	66.7	33.3	66.7
Strategy change from pre- to post-testing %	45				55.6			

It may be prudent to consider that this study examined independently living in older adults. This population may not stay in regular contact with gerontologists, who can educate on the importance of falls prevention for this cohort. In this target group, task managing training is an additional option for primary prevention of future falls. The group training sessions included daily circumstances that may lead to a fall (e.g., step over obstacles or a fast walking speed) and many participants were not sufficiently confident to manage these circumstances. While exercising with incrementally increased difficulty levels the participants learn, execute, and transfer these strategies during the training sessions. Future studies should document more precisely how task managing strategies change over the duration of a training period.

## Limitations

One limitation of the current study is the frequency of 1 dwk^-1^ of our training protocol; to improve balance the program should be conducted for a duration of 12 weeks with a frequency of three sessions per week with 31–45 min of training per session (Lesinski et al., 2015). We were not able to adopt this type of training protocol due to limitations dictated by the health insurance reimbursement to participants. This trial was conducted to examine the effects of task managing and resistance training interventions in a group of independently living healthy older adults. It is well documented, that motivating older adults to get more physically active has many barriers (e.g., Bethancourt et al., 2014). The health insurance reimbursement utilized in this study may have increased the motivation of the participants to join this study. Therefore, it is important to get the opportunity to work with health insurance funding to increase older individuals’ motivation to participate. While perhaps not as robust as possible with additional training sessions, both training groups displayed benefits in walking performance as a result of our training protocol.

A second limitation is that walking on a treadmill may not be comparable to over ground walking. However, some studies support the measurements used in this study during treadmill walking (Riley et al., 2007; Beurskens and Bock, 2013). To avoid confounding effects we spent much effort in the development of the test protocol (Wollesen et al., 2016). In addition, it is noteworthy to mention that the CG had a greater walking speed and associated step length. These differences in the intervention groups were observed, although the randomization process was stratified according to the SPBB and groups were not different after randomization. The significant group by measurement interaction revealed improvements in the DT experimental group that exceeded those of the CG, at least for gait-line in DT conditions, resulting in positive benefits of the intervention, especially the task managing training.

Nevertheless, a follow up study to determine effects of a longer training program as well as walking performance in a more complex balance environment may be useful to capture additional benefits of the two training interventions. Another limitation is that we did not control for the improvements of strength in walking-related muscle groups. This may need to be addressed in future studies.

## Conclusion

This randomized controlled trial allows for several conclusions associated with DT training interventions including task managing strategies. It was revealed that the active rolling movements of the foot increased in ST and DT conditions while COF decreased. Comparing the two training interventions (ST strength and resistance vs. BDT) the BDT training yielded greater improvements utilizing DT walking performance. We conclude that an improvement in walking performance and self-confidence during walking that is important for mobility and prevention of falls in older adults was achieved with the DT training. The length and evaluation of the program perhaps needs to be broadened to examine the effects on motor–cognitive performance in a more challenging balance environment as well as to include greater emphasis on task managing strategies of fallers to prioritize gait.

## Ethics Statement

The study was approved by the Ethics Committee of the Hamburg Chamber of Physicians (PV4376). All participants were informed about the study goals and risks, and signed informed consent prior to any testing according to the Declaration of Helsinki.

## Author Contributions

BW designed the study, interpreted the data and drafted the manuscript together with KM, SS, and LB organized the measurements and collected the data. LS organized and supervised the training sessions together with SS and helped editing the manuscript. BW, KM, and JB performed statistical analysis and review of the method section. SvD critically reviewed the manuscript and was responsible for the language editing.

## Conflict of Interest Statement

The authors declare that the research was conducted in the absence of any commercial or financial relationships that could be construed as a potential conflict of interest.
